# Pupil diameter as an indicator of sound pair familiarity after statistically structured auditory sequence

**DOI:** 10.1038/s41598-024-59302-1

**Published:** 2024-04-16

**Authors:** Janika Becker, Christoph W. Korn, Helen Blank

**Affiliations:** 1 Department of Systems Neuroscience, University Medical Center Hamburg-Eppendorf, Martinistr. 52, 20246 Hamburg, Germany; 2 Section Social Neuroscience, Department of General Psychiatry, University of Heidelberg, 69115 Heidelberg, Germany

**Keywords:** Perception, Sensory processing

## Abstract

Inspired by recent findings in the visual domain, we investigated whether the stimulus-evoked pupil dilation reflects temporal statistical regularities in sequences of auditory stimuli. We conducted two preregistered pupillometry experiments (experiment 1, *n* = 30, 21 females; experiment 2, *n* = 31, 22 females). In both experiments, human participants listened to sequences of spoken vowels in two conditions. In the first condition, the stimuli were presented in a random order and, in the second condition, the same stimuli were presented in a sequence structured in pairs. The second experiment replicated the first experiment with a modified timing and number of stimuli presented and without participants being informed about any sequence structure. The sound-evoked pupil dilation during a subsequent familiarity task indicated that participants learned the auditory vowel pairs of the structured condition. However, pupil diameter during the structured sequence did not differ according to the statistical regularity of the pair structure. This contrasts with similar visual studies, emphasizing the susceptibility of pupil effects during statistically structured sequences to experimental design settings in the auditory domain. In sum, our findings suggest that pupil diameter may serve as an indicator of sound pair familiarity but does not invariably respond to task-irrelevant transition probabilities of auditory sequences.

## Introduction

We constantly sample sensory information from our surroundings and update our inner model of the world based on the perceived relationships in our environment. The extraction of regularities such as the temporal co-occurrence of stimuli is thought to happen unconsciously and quickly, after only a few repetitions of the regularity^1,2^. Based on the perceived regularities, we can generate expectations about our environment. The violation of these expectations, potentially signalling a need for expectation updating, is experienced as surprise^3^ and linked to the neuromodulator norepinephrine (NE)^4–6^ which is generated in the Locus Coeruleus (LC). The LC-NE system is associated with the regulation of global arousal and projects to neural circuits modulating pupil diameter^7^. Consequently, pupillometry is often used as a proxy for global arousal^8–11^ and surprise-driven stimulus responses^3,10,12–16^.

Recent studies found stimulus-evoked pupil dilation to respond to transition probabilities in statistically structured visual sequences^12,17^, without participants being aware of any rule driving the stimulus presentation or being able to reproduce the statistical regularities. In these studies, participants were presented with sequences of visual stimuli in which the stimulus transitions were governed by different probabilities. In the study of Alamia et al.^12^, transitions between stimuli (four letters presented at 1 Hz) followed a probabilistic Markovian process, with some letter transitions occurring more frequently than others. The authors found a larger pupil dilation in response to rare letter transitions in comparison to more frequent transitions in the letter stream. Schwiedrzik and Sudmann^17^ presented participants initially with a randomly ordered sequence of 18 faces at a rate of 2 Hz, followed by the same faces presented in a sequence with a pair structure. While they showed pupil cycling at the stimulus presentation rate in both conditions, pupil diameter additionally was smaller in response to the first stimulus than to the second stimulus of a pair in the structured sequence.

**Figure 1:**
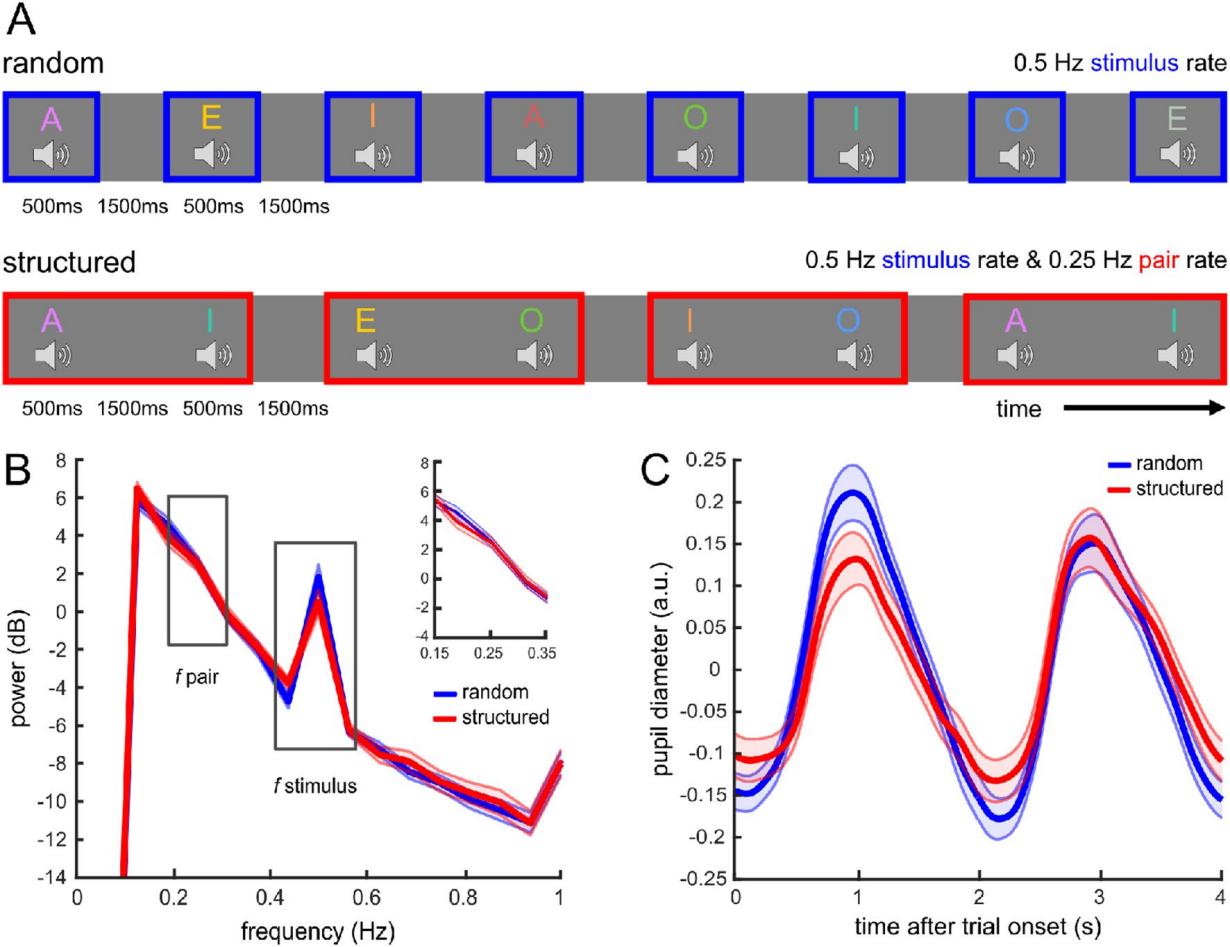
Design and main results of experiment 1. (**A**) Design of experiment 1. Participants were presented with auditory stimuli in two conditions: in the random condition, eight spoken vowels were presented in a random order with a stimulus presentation rate of 0.5 Hz, and afterwards, in the structured condition, the same stimuli were arranged in pairs, resulting in the pair presentation rate of 0.25 Hz. In this illustration, the colours of the vowels represent different voices. A white fixation cross was presented on the screen throughout the experiment. (**B**) Frequency power spectrum. The peak in spectral power at the stimulus presentation rate (0.5 Hz) is significant compared to the mean of the four surrounding frequency bins (*t*-tests, *p* < 0.001) in both the random and the structured condition, but there is no significant peak at the pair presentation rate (0.25 Hz, *p* > 0.05). (**C**) Mean pupil diameter for two consecutively presented stimuli (i.e., a ‘trial’) averaged over participants in the random (blue) and structured (red) condition (excluding trials with a task event and the following trial). The pupil dilates after sound onset (t = 0 s and t = 2 s) and peaks about one second later. Shading represents standard error of the mean (SEM) across participants.

As pupil dilation was also shown to respond to stimulus probability in the auditory domain^16,18–21^, we conducted two experiments to investigate whether pupil diameter indicates statistical regularities, as defined by stimulus transition probabilities, in auditory sequences. For this purpose, we transferred the visual study design by Schwiedrzik and Sudmann^17^ to the auditory domain. We opted for recordings of voices uttering different vowels to align our auditory stimuli with their visual stimuli consisting of face images with different head orientations. Matching their experiment structure, our participants were exposed to sequences of auditory stimuli in two conditions. In the first condition, the stimuli were presented in a randomized order. In the second condition, the same stimuli were temporally structured in pairs (Fig. 1A). In experiment 1, we presented eight voices with a stimulus presentation rate of 0.5 Hz and informed participants about the upcoming pair regularity in the structured sequence. As we could not reproduce the results from the visual domain using these settings, we conducted a second experiment that mirrored the design of the visual study (i.e., 18 face images presented at 2 Hz) more closely^17^. To this end, in experiment 2, we increased the number of voices to 18 and the stimulus presentation rate to 1 Hz (note that 2 Hz was not feasible due to the auditory stimulus duration), we did not inform participants about any statistical rules driving the stimulus sequences, and we conducted a familiarity task after the sequences to determine whether participants learned the pairs presented in the structured condition.

For the familiarity task, we found that pupil diameter following a stimulus pair differed depending on whether participants were exposed to that pair in the structured sequence or not, indicating that pupil diameter can serve as an indicator of learned auditory regularities. However, in both experiments, we did not find the expected pupil response to the transition probabilities during the presentation of the sequences. This observation points out that pupil effects in response to statistically structured sequences are susceptible to the specific experimental settings, potentially particularly in the auditory domain.

## Results

In two preregistered experiments, we analysed the pupil diameter of 30 (experiment 1) and 31 participants (experiment 2) who listened to auditory sequences of vowels spoken by different speakers. In both experiments, participants were first presented with the stimuli in a random order (random condition), and afterwards, with the same stimuli structured in pairs (structured condition). In the random condition, stimulus transition probabilities were equal between all stimuli, while in the structured condition, the stimulus transition probabilities depended on the pair structure of the sequence. Specifically, the stimulus transition probabilities within pairs were at maximum (i.e., voice ‘X’ was always followed by voice ‘Y’) and the stimulus transition probabilities between pairs were at minimum (i.e., stimulus pair XY could be followed by the pairs AB, CD, EF, or XY with the same probability).

**Figure 2:**
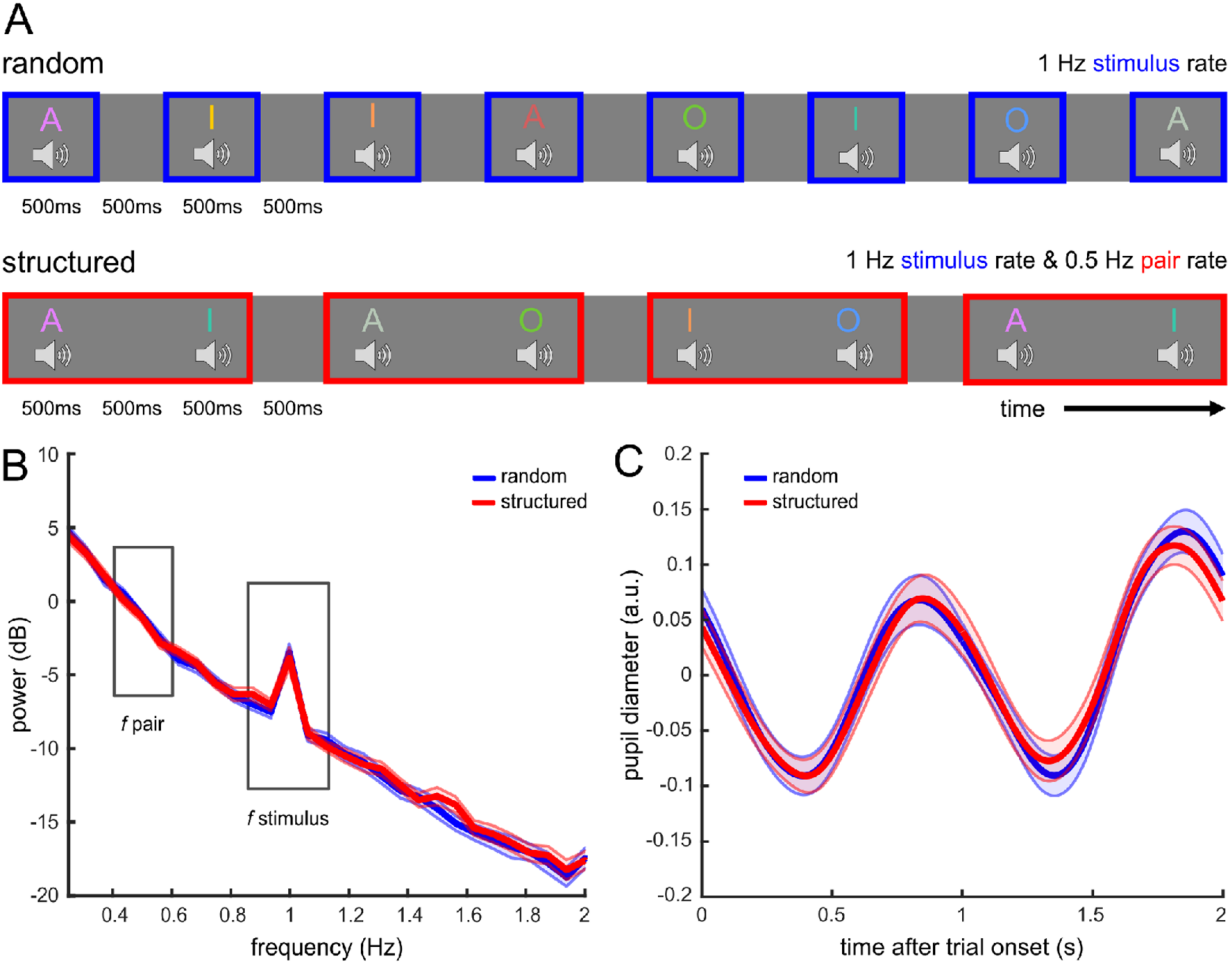
Design and main results of experiment 2. (**A**) Design of experiment 2. Participants listened to 18 different voice stimuli in the random and structured condition, presented at a stimulus presentation rate of 1 Hz. In the structured condition, the pair presentation rate was 0.5 Hz. (**B**) Frequency power spectrum. The power spectrum shows a significant peak in both conditions at the stimulus presentation rate (1 Hz, *p*s < 0.001), but no significant peak at the pair presentation rate (0.5 Hz, *p* > 0.05). (**C**) Pupil diameter locked to the onset of a trial for the random (blue) and the structured (red) condition. Shading represents SEM across participants.

In experiment 1, participants listened to sequences consisting of eight stimuli (8 voices, 4 vowels) presented at 0.5 Hz and had to respond to an infrequent visual task (i.e., a luminance change of the fixation cross) and auditory task (i.e., auditory 1-back repetition detection task) (Fig. 1A). Also, they were informed before the structured condition that auditory stimuli would be presented in pairs. In experiment 2, a new sample of participants listened to sequences consisting of 18 stimuli (18 voices, 3 vowels) presented at 1 Hz while responding to an infrequent auditory task (i.e., auditory 1-back repetition detection task) (Fig. 2A). They were not informed about any structure in the stimulus presentation. After listening to the stimulus sequences in experiment 2, participants completed a familiarity task to test whether they learned the stimulus pairs in the structured condition.

In both experiments, behavioural performance in the auditory 1-back task was high across the analysed data blocks (Exp. 1: *M* = 89.8%, *SD* = 6.5; Exp. 2: *M* = 78.7%, *SD* = 9.8). Yet we did not find the hypothesized pupil effects of the statistical pair structure during the auditory sequences. However, pupil diameter during the familiarity task after experiment 2 differed between familiar pairs, which participants were previously exposed to in the structured condition, and foil pairs, indicating that pupil diameter reflected the learned association of auditory stimuli.

### Pupil dilation reflects sound presentation but not statistical pair structure

We investigated whether the pupil dilation responses during the auditory sequences reflect, firstly, the stimulus presentation rate (i.e., dilation in response to every sound), and secondly, in the structured condition, also reflect the pair presentation rate (i.e., dilation in response to every sound pair). Consequently, we tested for two peaks in a frequency power spectrum by comparing the peak value to the mean of the four surrounding frequency bins: one peak at the stimulus presentation rate (Exp. 1: 0.5 Hz; Exp. 2: 1 Hz) in both conditions (random and structured) and an additional peak at the pair presentation rate in the structured condition only (Exp. 1: 0.25 Hz; Exp. 2: 0.5 Hz).

As expected, the frequency power spectra of both experiments showed a distinct peak at the stimulus presentation rate in both conditions (Exp. 1: random condition: *t*(29) = 10.318,* p* < 0.001, *d*_*z*_ = 1.88, BF_10_ > 100; structured condition, *t*(29) = 8.101, *p* < 0.001, *d*_*z*_ = 1.48, BF_10_ > 100; Exp. 2: random condition, *t*(30) = 9.349, *p* < 0.001, *d*_*z*_ = 1.679, BF_10_ > 100; structured condition, *t*(30) = 6.544, *p* < 0.001, *d*_*z*_ = 1.175, BF_10_ > 100, see Figs. 1B, 2B). However, there was no peak at the pair presentation rate in the structured conditions (Exp. 1: *t*(29) = 1.188, *p* = 0.122, *d*_*z*_ = 0.217, BF_10_ = 0.647; Exp. 2: *t*(30) = 0.163, *p* = 0.436, *d*_*z*_ = 0.029, BF_10_ = 0.219). Consequently, we neither found the hypothesized interaction of condition and frequency in a repeated-measures ANOVA (rmANOVA, Exp. 1: *F*(1, 29) = 4.093, *p* = 0.052, η_p_^2^ = 0.124, BF = 0.44; Exp. 2: *F*(1, 30) = 0.119, *p* = 0.733, η_p_^2^ = 0.004, BF = 0.166) nor a significant difference in power between the random and structured conditions at the pair presentation rate (Exp. 1: *t*(29) =  − 0.118, *p* = 0.546, *d*_*z*_ =  − 0.022, BF_10_ = 0.178; Exp. 2: *t*(30) =  − 0.387, *p* = 0.649, *d*_*z*_ =  − 0.07, BF_10_ = 0.144).

Additionally, we calculated the intertrial phase clustering (ITPC) to test pupil entrainment to the auditory stimulus and pair presentation. The ITPC across both conditions was higher at the stimulus presentation rate than at the pair presentation rate (Exp. 1: mean difference = 0.34, *t*(29) = 11.7, *p* < 0.001, *d*_*z*_ = 2.14, BF_10_ > 100; Exp. 2: mean difference = 0.26, *t*(30) = 7.23, *p* < 0.001, *d*_*z*_ = 1.3, BF_10_ > 100), showing that the pupil dilation tracked the auditory stimuli. However, there was no difference in ITPC at the pair presentation rate between the random and the structured condition (Exp. 1: mean difference = 0.01, *t*(29) = − 0.65, *p* = 0.52, *d*_*z*_ = − 0.12, BF_10_ = 0.23; Exp. 2: mean difference = 0.01, *t*(30) = 0.72, *p* = 0.48, *d*_*z*_ = 0.13, BF_10_ = 0.24), indicating that the pupil dilation did not entrain to the statistical structure of the sound pairs in the structured condition.

### Pupil diameter responds to sound presentation but not statistical pair structure

For complementary tests to the frequency power analyses, we extracted the pupil diameter at the onset of a trial. We defined a trial as spanning two consecutively presented stimuli including their following inter-stimulus interval (ISI), respectively (i.e., trial duration: Exp. 1: 4000 ms; Exp. 2: 2000 ms). In the structured condition, a trial consisted of the two stimuli composing a pair. In both experiments, pupil diameter peaked in the time window after each sound onset (Figs. 1C, 2C), corresponding to the peaks at the stimulus presentation rate in the frequency power spectra (Figs. 1B, 2B). In accordance with the frequency power analyses, maximum pupil diameter after sound onset (time window: Exp. 1: 0–2000 ms, Exp. 2: 200–1000 ms) did not show a significant interaction of condition and stimulus position within a trial (rmANOVA: Exp. 1: *F*(1, 29) = 3.269, *p* = 0.081, η_p_^2^ = 0.101, BF = 0.942; Exp. 2: *F*(1, 30) = 0.298, *p* = 0.589, η_p_^2^ = 0.01, BF = 0.187). Pupil diameter did not significantly differ between the random and structured condition (no main effect of condition, Exp. 1: *F*(1, 29) = 3.232, *p* = 0.083, η_p_^2^ = 0.1, BF = 0.61; Exp. 2: *F*(1, 30) = 0.724, *p* = 0.402, η_p_^2^ = 0.024, BF = 0.138). Also, pupil diameter did not significantly differ between the stimulus positions within a trial in experiment 1 (no main effect of stimulus position: *F*(1, 29) = 0.447, *p* = 0.509, η_p_^2^ = 0.015, BF = 0.155). In experiment 2, maximum pupil diameter was larger in response to every second stimulus in both conditions (main effect of stimulus position: *F*(1, 30) = 9.096, *p* = 0.005, η_p_^2^ = 0.233, BF = 112.036; pupil diameter after stimulus 1: *M* = 0.128 a.u., *SD* = 0.097, after stimulus 2: *M* = 0.176, *SD* = 0.08).

Next, we tested for an effect of the sequence structure on minimum pupil diameter during the ISI. In both experiments, minimum pupil diameter during the ISI (time window after sound onset: Exp. 1: 1000–3000 ms, Exp. 2: 700–1200 ms) did neither show an interaction of condition and ISI position within a trial (rmANOVA, Exp. 1: *F*(1, 29) = 0.599, *p* = 0.445, η_p_^2^ = 0.02, BF = 0.2; Exp. 2: *F*(1, 30) = 0.051, *p* = 0.822, η_p_^2^ = 0.002, BF = 0.184) nor a main effect of ISI position within a trial (Exp. 1: *F*(1, 29) = 0.453, *p* = 0.506, η_p_^2^ = 0.015, BF = 0.184; Exp. 2: *F*(1, 30) = 1.017, *p* = 0.321, η_p_^2^ = 0.033, BF = 0.234). In experiment 1, a significant main effect of condition (*F*(1, 29) = 6.446, *p* = 0.017, η_p_^2^ = 0.182, BF = 0.76) indicated that minimum pupil diameter was smaller in the random condition (*M* =  − 0.221 a.u., *SD* = 0.114) than in the structured condition (*M* =  − 0.194, *SD* = 0.114). There was no main effect of condition in experiment 2 (*F*(1, 30) = 0.333, *p* = 0.568, η_p_^2^ = 0.011, BF = 0.132).

Finally, we tested whether a differential pupil dilation response to the pair structure developed over time in the structured condition, i.e., whether the pupil dilation response to the stimuli of a pair differed between the beginning (first block) and the end (last block) of the structured condition. For both experiments, maximum pupil diameter showed no significant main effects or interaction, so there was neither a difference in pupil diameter between the first and the last block of the structured condition (rmANOVA, no main effect of block, Exp. 1: *F*(1, 20) = 3.394, *p* = 0.08, η_p_^2^ = 0.145, BF = 0.355; Exp. 2: *F*(1, 23) = 0.004, *p* = 0.952, η_p_^2^ < 0.01, BF = 0.152), nor in response to the first and second stimulus within a pair (no main effect of stimulus position, Exp. 1: *F*(1, 20) = 0.02, *p* = 0.89, η_p_^2^ < 0.001, BF = 0.147; Exp. 2: *F*(1, 23) = 2.286, *p* = 0.144, η_p_^2^ = 0.09, BF = 0.878), and no significant interaction of block and stimulus position within a pair (Exp. 1: *F*(1, 20) = 2.147, *p* = 0.158, η_p_^2^ = 0.097, BF = 0.386; Exp. 2: *F*(1, 23) = 1.9, *p* = 0.181, η_p_^2^ = 0.076, BF = 0.467).

### Familiarity task: pupil diameter differs between familiar and foil pairs

**Figure 3:**
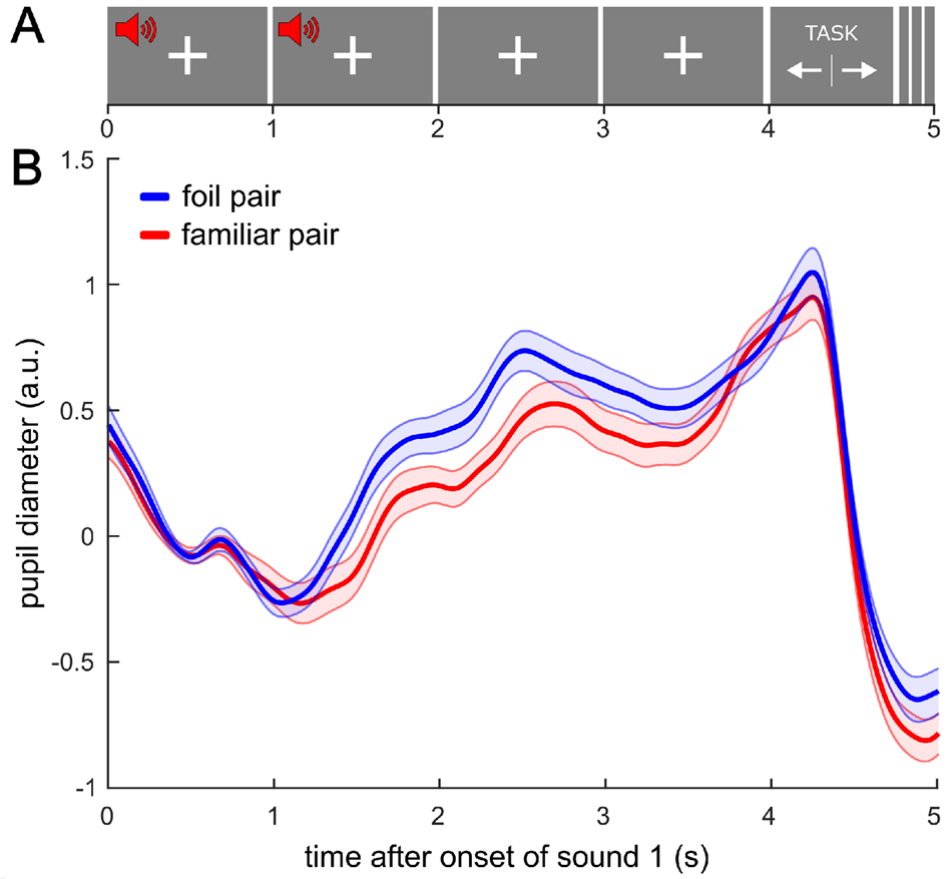
Pupil diameter during the familiarity task (familiar vs. foil pairs). (**A**) Participants heard either a stimulus pair they were exposed to during the structured sequence (‘familiar pair’) or a new combination of stimuli (‘foil pair’). Stimulus 1 was presented at t = 0 s and stimulus 2 at t = 1 s. At t = 4 s, the choice options were presented onscreen and participants had to indicate via button press whether the presented pair was familiar or unfamiliar to them. (**B**) Maximum pupil diameter in the two-second window after the onset of stimulus 2 was larger for foil pairs, indicating that pupil diameter reflected implicit learning of the pairs in the structured condition. Shading represents SEM across participants.

After experiment 2, participants completed a familiarity task in which they were presented with ‘familiar’ stimulus pairs, which were part of the previously presented structured condition, and ‘foil’ pairs (i.e., pairs consisting of the first stimulus of one pair and the second stimulus of another pair), which were not presented in the structured condition. After the pair presentation, participants had to indicate via keypress whether the pair was familiar or unfamiliar (Fig. 3A). Behavioural measures did not indicate that participants learned the pairs, as accuracy was not significantly above chance (*M* = 52% rated correctly, *SD* = 9, range = 28–67; *t*(30) = 1.452, *p* = 0.157, *d*_*z*_ = 0.261, BF_10_ = 0.495) and reaction time did not differ between familiar (*M* = 1.051 s, *SD* = 0.583) and foil pairs (*M* = 1.135 s, *SD* = 0.522, *t*(30) =  − 0.926, *p* = 0.362, *d*_*z*_ =  − 0.166, BF_10_ = 0.284). However, maximum pupil diameter after the pair presentation differed significantly between familiar and foil pairs (*t*(30) = 2.735, *p* = 0.01, *d*_*z*_ = 0.491, BF_10_ = 4.32), with a larger pupil diameter after foil pairs (*M* = 0.958, *SD* = 0.366) than after familiar pairs (*M* = 0.728, *SD* = 0.377, Fig. 3B). We explored whether participants who performed better in the familiarity task also showed a stronger differential pupil response and found no correlation of the mean difference in maximum pupil diameter between foil and familiar pairs with the participants’ task accuracy (*r* = 0.29, *p* = 0.11).

Additionally, a linear mixed-effects model showed that maximum pupil diameter was best explained by the true pair category (*β* = 0.198, *t*(554) = 2.19, *p* = 0.029), while the estimated pair category as indicated by the participants’ response (*β* =  − 0.126, *t*(554) =  − 1.38, *p* = 0.167) and the reaction time (*β* = 0.016, *t*(554) = 0.4, *p* = 0.684) did not significantly contribute to the maximum pupil diameter. The positive estimate of the predictor ‘true category’ supports the previous finding of a larger pupil diameter in response to foil pairs, whereby the pupil diameter reflects the actual rather than the reported pair category.

In sum, we did not observe an effect of the statistical pair structure on the pupil response during the presentation of the auditory sequences. However, we found that maximum pupil diameter in a subsequent familiarity task distinguished between the stimulus pairs of the structured condition and foil pairs, indicating that participants learned the pairs.

## Discussion

In this study, we tested whether the stimulus-evoked pupil dilation during auditory sequences differs depending on the statistical structure of the sequence. In contrast to previous observations of pupil diameter reflecting differences in the statistical structure of visual sequences of letters or faces^12,17^, we did not find corresponding pupil effects during auditory sequences of spoken vowels. However, in a subsequent familiarity task, pupil diameter differed between familiar and foil stimulus pairs. We speculate that the extent to which the stimulus-evoked pupil dilation response in the auditory domain reflects learned statistical regularities, based on stimulus transition probabilities, depends on factors such as the sequence complexity, stimulus distinctiveness, and relevance of the regularities to the task at hand.

We conducted two preregistered pupillometry experiments in which participants were exposed to sequences of auditory stimuli (vowels spoken by different speakers). In the ‘random’ condition, the stimuli were presented in a random order. Afterwards, in the ‘structured’ condition, the same stimuli were temporally grouped in pairs, such that every second stimulus was predictable based on its predecessor. Participants performed an infrequently and randomly occurring 1-back auditory repetition detection task. In experiment 1, participants listened to 8 stimuli (i.e., 8 different speakers uttering one of 4 vowels) at a presentation rate of 0.5 Hz. As a recent study^16^ found that some participants only exhibited pupil dilation effects in response to violations of global regularity after they were explicitly made aware of the regularity, we informed participants about the pair structure before the start of the structured sequence. For experiment 2, we adapted our experimental design to increase the similarity to the main experiment of Schwiedrzik and Sudmann^17^. For this purpose, we increased the number of auditory stimuli from 8 to 18 (i.e., 18 different speaker-vowel combinations) as well as the stimulus presentation rate (from 0.5 to 1 Hz) and we did not inform participants about the sequence structure. To assess learning of the stimulus pairs in experiment 2, participants also completed a familiarity task after being exposed to the sequences.

During the familiarity task, the pupil diameter was smaller following familiar pairs, i.e., sound pairs which participants were previously exposed to in the structured condition, in comparison to foil pairs, i.e., new pair combinations of the stimuli. This finding, supported by the results of a linear mixed-effects model, suggests that pupil diameter provides a valuable measure to assess implicitly learned stimulus associations, provided that the stimuli are made behaviourally relevant to the listeners. In contrast to the differential pupil response, the behavioural results, i.e., reaction time and accuracy in rating pairs as familiar or unfamiliar, did not indicate that participants explicitly learned the auditory stimulus pairs. This finding is in line with previous studies, where behavioural measures of rule awareness similarly did not indicate that participants had learned the statistical patterns underlying the sequences. (i.e., frequent letter transitions or face pairs, see Alamia et al.^12^, experiment 2, familiarity and generative task, and Schwiedrzik and Sudmann^17^, experiment 2, card sorting task). In our experimental design, however, the usefulness of reaction time as a measure of learning is constrained due to the delayed response option, allowing participants to prepare their answers in advance.

Our study supports previous studies challenging the broad applicability of pupil diameter as an indicator of statistical learning, emphasizing the need for cautious interpretation^22^. However, we cannot conclude from the absence of pupil dilation in response to statistical regularities in our study that the pupil does not reflect auditory statistical regularities in general. More sensitive measures, such as electroencephalography^23–25^, or additional indicators of ocular activity^26^, such as electrooculogram, saccades or blinks, may be needed to detect an auditory-regularity effect in both the exposure phase and the familiarity task. In the following sections, we discuss potential reasons for the absence of the expected pupil effect of the pair structure during the auditory sequences.

As the effects of previous visual studies did not easily translate to the auditory domain, the sensory modality might be considered a critical factor for pupil effects of statistical structures during stimulus sequences. The visual and the auditory domain are based on inherently different environmental statistics, such that visual stimuli are more stable and hence changes are more surprising than in the constantly dynamic auditory domain, so there might be modality-specific mechanisms of processing temporal statistical regularities^27,28^. However, there is a body of existing research using pupillometry and auditory stimuli to investigate the processing of probabilities and predictability. For example, sound-evoked (phasic) pupil dilation differed depending on the stimulus occurrence probability^18–21^. Also, sustained (tonic) pupil diameter differed depending on the statistical structure of an auditory sequence^1^. Milne et al.^1^ found larger sustained pupil dilation during random auditory sequences than during sequences with a probabilistic or deterministic structure, possibly indicating that predictability facilitated sequence processing in the structured sequences. Generally, auditory processing is thought to have a higher temporal sensitivity than visual processing^27,29,30^ and an advantage in sequential processing^28,31^. Therefore, as previous studies showed pupil effects of predictability with auditory stimuli, the modality alone does not appear to account for the lack of pupil effects during the auditory sequences in our study.

A second distinction between our study and previous visual research was the presentation rate. Due to the auditory stimulus length of 0.5 s, the stimulus presentation rate in our experiment 2 (1 Hz) was lower than the presentation rate of the visual stimuli (2 Hz) used by Schwiedrzik and Sudmann^17^, but the same as used by Alamia et al.^12^ who also reported pupil effects of stimulus transition probabilities in a visual sequence. Furthermore, previous auditory studies with stimulus presentation rates comparable to ours found predictability-related differences in the pupil response to sound sequences. For example, Qiyuan et al.^19^ presented auditory sequences of 50-ms tones at 1.5-s intervals (i.e., 0.666 Hz stimulus presentation rate) and reported that the amplitude of the sound-evoked pupil dilation was modulated by tone probability. Predictability-related pupil effects were also observed in musical research, e.g., by Bianco et al.^32^ who used 5-s sequences of 20 tones (i.e., 4 Hz stimulus presentation rate) and found a difference in the pupil dilation response between expected and unexpected tones. Although their paradigm included deviants and, thus, is not directly comparable to our paradigm, it shows that predictability-related effects are observable in the pupil response to auditory stimuli with an even higher presentation rate. Therefore, it is unlikely that the absence of probability-related pupil effects in our study is due to the chosen stimulus presentation rates in the sequences.

A dissimilarity between our study and the majority of previous auditory studies is the specific choice of stimuli, as previous studies predominantly used tones^1,19,20,32,33^, whereas we used vowels spoken by different speakers. However, some studies have successfully used verbal auditory stimuli to investigate statistical learning based on transition probabilities in sequences^34^, especially in the field of language acquisition^35^. In a seminal study, Saffran et al.^36^ showed that 8-month-old infants learned the transition probabilities of auditory syllables (i.e., consonants followed by a vowel) within a short period of time. In contrast to their experiment and the large body of studies replicating this effect, our stimulus material consisted of multiple speakers and contained only vowels. According to the consonant–vowel hypothesis^37,38^, humans extract words in a continuous artificial stream of speech primarily based on the statistical dependencies of consonants, not vowels. Therefore, the usage of only vowels in our study might have hampered statistical learning of the transition probabilities, even though the familiarity task indicated that participants were able to learn the vowel pairs. Consequently, our paradigm is not fully comparable to the mentioned studies in the domain of language acquisition.

Furthermore, our stimulus material consisted of voice recordings from multiple speakers who naturally differed in their voice characteristics and, thus, potentially in saliency. The stimuli were adjusted to have equal duration and loudness, while intentionally varying in frequency-related features, such as pitch and formant frequencies, as these are crucial for defining voice and vowel identity^39,40^. Our stimuli were derived from the natural spectrum of frequency variations rather than artificially generated, so any potential differences in saliency are inherently embedded within the defining features of our stimuli. As saliency draws attention^41^ and the pupil reflects temporal attention^26^, a variation in stimulus-related saliency could have added noise to the observed stimulus-evoked pupil dilation in our study, potentially obscuring a regularity-related effect. Yet, given the balanced stimulus sets over participants and the randomized order of stimuli and pairs in the sequences, any saliency-related pupil responses to single stimuli should be cancelled out during the calculation of intertrial phase clustering or frequency power over the entire sequences. Overall, this supports the conclusion that pupil diameter can reflect statistical regularities but might not be a broadly applicable indicator of regularities in sequences with diverse stimuli.

As fatigue or habituation effects are known to be reflected in the pupil dilation response^42^, these might also affect the pupil responses in our experimental design. For example, in experiment 1, minimum pupil diameter was smaller between stimuli in the random condition than in the structured condition, although the effect was not supported by the related Bayes Factor. As the random condition was presented first, it was probably less affected by fatigue or habituation effects, so the pupil diameter amplitude was larger. Nevertheless, it is unlikely that potential regularity-related pupil effects were completely masked by fatigue as the duration of our experiments and the length of blocks are comparable to other sequence experiments that found regularity-related pupil effects in the visual^12,17^ or auditory domain^1^.

Moreover, although research indicates that statistical learning can happen automatically, rapidly, and unconsciously^2^, studies suggesting that statistical learning is task-independent also observed that pupil effects of statistical learning disappeared when attention was diverted from the statistically structured stimuli^12,43^. We included the auditory 1-back repetition task in both experiments to increase participants’ engagement and for comparability to the study by Schwiedrzik and Sudmann^17^. As the temporal statistical structure of the sequence was critical for the 1-back repetition task, we assumed that participants paid attention to the temporal structure of the sequence. To prevent any unintended shifts of attention by assigning tasks in two distinct modalities in our first experiment, we removed the visual task in the second experiment. It is unclear whether the 1-back task affected learning of the statistical structure in our experiments: the task could have facilitated the processing of the pair structure or could have disrupted the emerging pair structure by requiring participants to direct their attention to the stimulus repetitions. Unexpected auditory repetitions, i.e., infrequent repetitions of the same stimulus in a sequence of alternating stimuli^44^, have been found to draw attention even when to be ignored during a visual memory task^45^ and elicit a pupil dilation at first occurrence^6,46^. Hence in our design, participants just needed to react to the attention-drawing violation of the basic regularity of stimulus alternation and could have neglected the pair structure. However, the difference in the pupil response to familiar and foil pairs in the familiarity task indicates that participants processed the pair regularity of the structured sequence at least implicitly.

As a final side note, in experiment 2, we observed a larger pupil diameter in response to every second stimulus in both the random and the structured condition. As there was no statistical structure in the random condition, which the pupil might have responded to, we report this unexpected finding and cautiously interpret this as a coincidental additional occurrence of pupil cycling at 0.5 Hz to the auditory stimuli presented at 1 Hz. Further studies are needed to find out whether this observation is a false positive result, as it is not evident in the corresponding frequency power spectrum, or whether it indeed reflects some kind of automatic pupil cycling at 0.5 Hz during random auditory sequences (potentially in relation to fatigue effects^47^ or bottom-up effects of peripheral autonomic function^48^).

In summary, there are several differences between our study design and previous studies, which showed differential pupil responses to regularities in visual sequences as well as to auditory stimuli, that may account for the absent pupil response to the pair structure in our auditory sequences. The pupil effect in our subsequent familiarity task suggests that the behavioural relevance of statistical regularities might be crucial to observe a transition probability-related stimulus-evoked pupil dilation in the auditory domain. Previous visual studies found pupillometry to be a promising method for monitoring the acquisition of sensory statistical regularities in sequences online^12,17^, potentially able to replace explicit questioning or subsequent testing of learning success in the form of familiarity or generative tasks. However, our study questions the generalizability of this effect, indicating that this might not readily apply to any sensory sequence and may depend on the interplay of the specific experimental settings, such as the modality, stimulus characteristics, timing or task relevance and difficulty.

The pupil dilation response during the explicit familiarity task indicated that participants learned auditory pairs in the structured condition, but pupil diameter during the structured sequence did not vary with the statistical regularity in our study. In conclusion, pupil diameter may indicate sound pair familiarity but may not universally respond to task-irrelevant transition probabilities in auditory sequences.

## Materials and methods

### General procedure

In two preregistered pupillometry experiments, participants listened to auditory sequences. The pupillometry measurements took place in a darkened room (experiment 1) and with a dim light behind the participants (experiment 2). Participants placed their heads on a chin support to ensure head stability and a constant distance of 50 cm from the monitor. Using an EyeLink 1000 eye tracker, the gaze and diameter of the participants’ right pupils were recorded at a sampling rate of 1000 Hz. The experiment was implemented in Matlab (version 2013b^49^). Auditory stimuli were presented via headphones (Sennheiser HD 201) and button presses were recorded with a conventional PC keyboard.

The auditory sequences consisted of single vowels spoken by different speakers. The sequences were presented in two conditions which differed only in the statistical structure of the stimulus presentation^17^: first, in the random condition, the stimuli were presented in a randomized order. Afterwards, in the structured condition, the same stimuli were temporally arranged in fixed pairs, such that the first stimulus of a pair predicted the subsequently presented second stimulus of that pair. The stimulus pairs were presented in a randomized order. Participants always took part in the random condition first and in the structured condition afterwards. This was to ensure that participants did not have any pair-related expectations about the stimulus presentation structure in the random condition which could have affected the pupil response.

#### Ethics Statement

Both experiments were approved by the Ethics Committee of the Chamber of Physicians in Hamburg and were performed in accordance with the ethical guidelines and regulations. All participants gave written informed consent to privacy terms and conditions before they participated in the study. Both experiments were preregistered before any data analyses were conducted (Exp. 1: https://osf.io/d8utw, Exp. 2: https://osf.io/8gfn5).

### Experiment 1

#### Participants

A total of *N* = 38 participants were measured for experiment 1. Eight data sets were excluded from the analysis (six sets met the predefined exclusion criteria related to task performance and missing pupil data proportion, two others were excluded due to technical failure during the measurement and the raw visualized data exhibiting large artefacts). Thus, the data sets of a final *n* = 30 participants (21 females and 9 males; *M* = 26.2 years, *SD* = 4.0, range 18–33) were used for further analyses.

Using the software G*Power^50^, the preregistered sample size was estimated with a power analysis based on the effect size reported by Schwiedrzik and Sudmann^17^ for the difference in spectral power at the pair presentation rate between the structured and random condition: *t*(29) = 3.451; *p* = 0.002; *Hedges g* = 0.449, which was transformed to *d*_*z*_ using the formula Cohen’s *d*_*z*_ = $\frac{t}{\sqrt{n}}$^51^. Aiming for 0.90 power to achieve an effect size of *d*_*z*_ = 0.63 with an alpha error probability of 0.05, the power analysis yielded a target sample size of *N* = 29. As we invited more participants to prospectively compensate for data set exclusion, we ended with full data sets of 30 participants. Participants were recruited via the university job portal and received monetary compensation for their participation. Participants were required to have very good German language skills, be between 18 and 35 years old and physically and mentally healthy. Wearing glasses or contact lenses as well as regular use of medication or other intoxicants were considered exclusion criteria. The participants were asked to refrain from wearing heavy eye makeup and from consuming caffeine before the measurement.

#### Stimuli, apparatus, and procedure

For our study, we aimed at auditory stimuli that resembled the visual stimuli used in the study by Schwiedrzik and Sudmann^17^. They presented 18 images of different faces with one of three head orientations. To maintain the human character of the stimuli, we decided on voices as stimuli, and to account for the varying head orientation, we let the voices speak different vowels. The extent of similarity between our auditory and their visual stimuli was not quantified.

For our experiment 1, we wanted to facilitate learning of the stimuli and thus reduced the complexity of the sequence by presenting *n* = 8 voices. To balance the mapping of the vowels to the voices, we decided on *n* = 4 different spoken vowels (/a/, /e/, /i/, and /o/). We recorded eight different speakers (4 females, 4 males) of different heights (167–193 cm) and ages (20–55 years) with the microphone TLM 103 by Neumann. The recordings were standardized in volume (75 dB) and length (duration: 0.5 s) with version 2.4.2 of Audacity recording and editing software^52^. The voice characteristics, i.e., formant shift ratio and median pitch, of some speakers were slightly altered using the program Praat^53^ to create comparable but distinct voice stimuli. We created a pool of 32 stimuli, with eight voices speaking four vowels, from which we drew a pseudorandomized set of eight stimuli for every participant. It was predefined that every set contained all eight voices (i.e., every voice once) and all four vowels (i.e., every vowel twice). Thus, each participant heard eight different voices, with two voices speaking the same vowel. The specific voice-vowel combinations were pseudorandomly selected for every participant.

#### Experimental design

In experiment 1, each of the two conditions (random and structured) was about 20 min long and consisted of four blocks with 152 stimulus presentations each. An auditory stimulus was presented every 2 s, i.e., the stimulus presentation rate was 0.5 Hz. Consequently, the pair presentation rate in the structured condition was 0.25 Hz^17^ (Fig. 1B). We chose this timing for the stimulus presentation because we found the sound-evoked pupil dilation response to be slow in a previous experiment^21^, and similar observations were reported elsewhere^54,55^.

For the statistical analyses, we artificially segmented the stimulus sequences in ‘trials’, whereby we defined a trial as two consecutively presented stimuli and their following ISI. In the random condition, the stimuli were presented in a randomized order, thus, the transition probabilities *p* for the two stimuli of a trial were the same (*p* = 0.14). In the structured condition, the pair structure of the sequence established different transition probabilities within a trial: while the stimulus transition probability within a pair was maximal (*p* = 1), the transition probability between pairs was minimal (*p* = 0.25). It was predetermined that no stimulus would be presented twice in a row (unless it was a task trial, see below) or three times in a row.

One trial (i.e., two stimuli of 500 ms duration, followed by an ISI of 1500 ms each) had a total duration of 4 s. One block consisted of 76 trials with 152 auditory stimulus presentations and a total duration of 304 s. There was a break of 20 s between each of the four blocks within each condition and a 5-min break between the two conditions. Before the main experiment, participants completed a two-minute training session to get used to the task and the experimental conditions.

Participants had one visual and one auditory task during the experiment to ensure that they paid attention to the auditory stimuli while keeping their eyes open for eye tracking. The auditory task was a 1-back repetition detection task, so participants were asked to press a button whenever the same stimulus (i.e., voice-vowel combination) was presented twice in a row. For the visual task, participants were instructed to press a button in response to a subtle luminance change of the fixation cross (1000 ms) on the screen. Both tasks occurred infrequently and randomly. About 20% of the 76 trials per block contained a task, with half of them being auditory task trials and the other half being visual task trials. The fixation-cross change (i.e., visual task) occurred only during the ISI and, in the structured condition, occurred equally within and between stimulus pairs. Each of the eight auditory stimuli was used for the 1-back repetition task at least once per block. There was a maximum of one task per trial. As explicit knowledge supports the prediction of upcoming stimuli^56^, we informed participants in experiment 1 before the start of the structured condition that the sequence would be structured in pairs.

#### Preprocessing

All preprocessing and data analyses were conducted in Matlab (version 2019b and 2021b^49^) using the publicly available Matlab toolbox PsPM (version 5.0.0^57^).

First, the raw data transmitted by the eye tracker were split into data per block. In a second step, outliers were excluded, which were defined based on gaze. As a change in gaze direction may affect the measurement of pupil diameter^58,59^, pupil diameter data points were excluded whenever the respective data points of gaze exceeded the range of four degrees visual angle around the centre of the fixation cross.

Data blocks were excluded when more than 33% of the pupil diameter data were missing after outlier exclusion or when participants missed more than 30% of the auditory task. Based on these criteria, we excluded an average of *M* = 1.4 blocks (*SD* = 1.57, median = 1, range: 0–4) per participant in experiment 1. If more than two blocks per condition were excluded, the entire data set was excluded and replaced with the data set of a new participant. Furthermore, all raw data were visualized and inspected to check for poor data quality. This would be indicated by an abnormal distribution of the power spectra, by large remaining artefacts in the preprocessed data or by other striking anomalies in the visualized data. As stated previously, bad data quality was clearly visible in one data set, which was excluded from further analyses. Gaps in pupil diameter data, due to blinks or outlier exclusion, were closed using a shape-preserving interpolation method (Piecewise Cubic Hermite Interpolating Polynomial, Pchip^60^). Then, filters were applied to remove slow drifts and smooth the data. First, we constructed a low-pass filter at 5 Hz similar to the one used by Schwiedrzik and Sudmann^17^, as a one-pass/zero-phase, Kaiser-windowed sinc finite impulse response filter (filter order = 2677; transition width = 2.0 Hz; pass band = 0–4.0 Hz; stop band = 6–500 Hz; maximal pass band deviation = 0.0010 (0.10%); stop band attenuation =  − 60 dB^61^).

Initially, as noted in the preregistration, a baseline subtraction per block as well as detrending of the data were planned. However, after data collection, the subtraction of a valid baseline turned out to be unfeasible, because a lot of participants kept their eyes closed in the breaks between the blocks which left no data to draw a baseline from. So instead a two-pass/zero-phase, Butterworth infinite impulse response high-pass filter at 0.1 Hz was applied (filter order = 11; transition width = 0.05 Hz; pass band = 0.1–500 Hz; stop band attenuation =  − 60 dB^61^). In a last preprocessing step, the sampling rate was reduced to 500 Hz. As preregistered, we z-standardized all pupil data to account for inter-individual differences in pupil amplitude.

#### Statistical analyses

The analysis steps are for the most part adopted from the methods described by Schwiedrzik and Sudmann^17^. We expected the pupil to visibly dilate after the presentation of an auditory stimulus in both conditions. Crucially, in the structured condition, we expected the pupil to react differently to each of the two stimuli composing a pair due to the different transition probabilities.

To substantiate the assumption of pupil dilation following auditory stimuli, we tested whether the pupil dilates at the stimulus presentation rate of 0.5 Hz in both conditions. For this purpose, we performed a spectral power analysis. A discrete Fourier transform (DFT) of the pupil data was calculated per block, applying Welch’s method^62^ as implemented in the Matlab function *pwelch.m*. In order to obtain a spectral resolution of 0.0625 Hz, a Hanning window of 50,820 points (one-third of a block length), 16,940 overlapping points (one-third of the window length), and 8000 DFT points were used. Then, the power spectra were converted to decibels by taking the decadic logarithm and multiplying by 10. After averaging the remaining power spectra over blocks per participant, we computed a mean power spectrum over all participants per condition.

We expected a spectral peak at the stimulus presentation rate (i.e., 0.5 Hz) in the power spectra of both conditions. To statistically test the existence of such, we compared spectral power at 0.5 Hz to the mean of the four surrounding frequency bins (i.e., two above and two below), by means of a one-tailed paired *t*-test for both conditions, respectively. In the structured condition, we additionally expected a spectral peak at the pair presentation rate (i.e., 0.25 Hz), which was also tested by comparing spectral power at 0.25 Hz to the mean of the four surrounding frequency bins (two above, two below) in the structured condition only, using a one-tailed paired *t*-test. Then, we tested whether spectral power at the pair rate was significantly higher in the structured condition than in the random conditions with a one-tailed paired *t*-test. As preregistered and based on the hypothesized results of the former analyses, we conducted a two-way repeated measures ANOVA (rmANOVA) of spectral power with frequency (2 levels: 0.25 Hz, 0.5 Hz) and condition (2 levels: structured, random) as within-subject factors which we expected to show an interaction of frequency and condition.

For all rmANOVA and paired *t-*tests of pupil diameter, we defined the pupil response to a stimulus as the maximum pupil diameter in the 2000-ms time window after stimulus onset. To calculate maximum and minimum pupil diameter, we averaged pupil diameter during a four-second trial per participant over all included trials per condition (for the analyses with factor condition) or block (for the analyses with factor block). For the analyses of pupil diameter, we included only trials that did not contain a task and neither were preceded by a task trial, as rare events cause massive and several seconds long-lasting pupil dilations^12^. Excluding every task trial and their subsequent trial resulted in the exclusion of an average of *M* = 27.46 trials (range: 22–32) out of 76 trials per block per participant in experiment 1. Considering the relatively high percentage of excluded trials, we reduced the proportion of task trials in experiment 2 by increasing the overall amount of trials and removing the visual task.

To test whether maximum pupil diameter following the stimuli was different between conditions and if this difference was specific to the stimulus position within a trial (which defines the stimulus probability), a two-way rmANOVA of maximum pupil diameter was conducted with the within-subject factors condition (2 levels: structured, random) and stimulus position (2 levels: first stimulus, second stimulus).

Based on the results of Schwiedrzik and Sudmann^17^, we tested whether minimum pupil diameter following the ISI within pairs differed from minimum pupil diameter following the ISI between pairs in the structured condition (Fig. 1C). For this purpose, we conducted a two-way rmANOVA of pupil diameter following an ISI with condition (2 levels: structured, random) and position of the ISI within a trial (2 levels: first ISI, second ISI) as within-subject factors. Pupil diameter following an ISI was defined as the minimum pupil diameter in the time window from 1000 to 3000 ms after stimulus onset.

Furthermore, we wanted to determine whether pupil diameter following the two stimuli of a pair in the structured condition changed over time, as participants learned the pairs and thereby the associated stimulus probabilities. To test this, pupil dimeter following the first and second stimulus of a pair was compared between the first and last block of the structured condition. For this purpose, a two-way rmANOVA of pupil diameter following a stimulus was performed with block (2 levels: first block, last block) and stimulus position within a pair (2 levels: first position, second position) as within-subject factors.

To test whether the pupil dilation entrained to the auditory stimuli presented at 0.5 Hz and to the pairs presented at 0.25 Hz in the structured condition, we calculated intertrial phase clustering (ITPC)^17,63^. ITPC indicates the consistency of an oscillation’s phase angle across trials. Possible ITPC values range from a minimum of 0, when all phase angles are uniformly distributed, to a maximum of 1, when phase angles are identical across trials. To calculate ITPC, the preprocessed pupil data were divided into pseudo trials of 16 s, starting with the onset of the first stimulus of a pair (the timing was chosen based on the procedure in the visual study^17^). Using the Matlab toolbox Fieldtrip (version 20240214^64^), we performed a Fourier transform for each pseudo trial per block and participant (tapers: discrete prolate spheroidal sequences (DPSSs); spectral resolution: 0.0625 Hz; minimal spectral smoothing), resulting in a range of complex numbers. ITPC was computed with these complex numbers and the formula:

$$ITPC=\left|{n}^{-1}{\sum }_{r=1}^{n}{e}^{{ik}_{fr}}\right|$$

for *n* trials with the phase angle *k* on trial *r* at the frequency *f*. We calculated the mean ITPC per participant and condition across included blocks at the stimulus and pair presentation rate and conducted statistical comparisons between conditions and rates using paired *t*-tests.

The significance of analyses was assumed based on the standard *p* < 0.05 criterion. Conducted tests of difference were two-tailed unless stated otherwise. As effect sizes, we report partial eta squared for rmANOVA and Cohen’s *d*_*z*_ for paired *t-*tests^51^. Also, we report Bayes Factors for all analyses. Bayes Factors of main factors and interactions in rmANOVA are computed by dividing the Bayes Factor of the full model by the restricted model not including the respective main effect or interaction.

### Experiment 2

We conducted a second experiment with adjusted settings to make the design more similar to the one of Schwiedrzik and Sudmann^17^. The methods of experiment 2 correspond to experiment 1 if not stated otherwise in the following.

#### Participants

A new sample of *N* = 35 participants was tested for experiment 2. Five data sets were excluded from the analysis based on the predefined exclusion criteria relating to task performance and missing pupil data proportion. Thus, the data sets of a final *n* = 31 participants (22 females and 9 males; *M* = 27.7 years, *SD* = 4.9, range 20–38) were used for further analyses. The sample size was based on experiment 1 and the sample size reported by Schwiedrzik and Sudmann^17^. For experiment 2, we included healthy participants with corrected-to-normal vision using soft contact lenses.

#### Stimuli, apparatus, and procedure

For experiment 2, we adjusted and extended the stimulus pool from experiment 1. To more closely relate to the original study by Schwiedrzik and Sudmann^17^ using images of 18 face identities with three head orientations, we used 18 voices and three vowels (i.e., /a/, /i/, and /o/). To this end, we recorded 10 more voices (5 females, 5 males) of different height (160–199 cm) and age (25–59 years) with the same microphone as in experiment 1. A total of 18 voices speaking 3 vowels resulted in a pool of 54 stimuli. Each participant listened to a pseudorandomized set of 18 stimuli. We predetermined that all 18 voices were included in a set and that the number of vowels was balanced. As we had three different vowels, every vowel appeared six times in a set. The mapping of vowels and the speakers’ gender was also balanced, thus, every vowel was spoken by three male and three female voices.

#### Experimental design

To make the experimental design more similar to the timing of the main experiment by Schwiedrzik and Sudmann^17^, we increased the stimulus presentation rate for our experiment 2, i.e., the stimulus presentation rate was 1 Hz and the pair presentation rate in the structured condition was 0.5 Hz (Fig. 2B). Both conditions were about 11 min long and consisted of four blocks with 162 stimulus presentations each. An auditory stimulus was presented every second, so one trial (i.e., two stimuli of 500 ms duration, followed by an ISI of 500 ms each) had a total duration of 2 s. One block consisted of 81 trials with a total duration of 162 s. There was a break of 10 s between each of the four blocks of each condition and about 5 min break between the two conditions. Before the experiment, participants completed a two-minute training session to get used to the task and the experimental conditions.

To make experiment 2 more similar to Schwiedrzik and Sudmann^17^, participants were only assigned with the auditory 1-back task, equivalent to their visual 1-back task. About 11% of the 81 trials per block were task trials. Per condition, each of the 18 auditory stimuli comprised the task trial at least twice. Task trials occurred randomly in the sequence but task trials did not appear consecutively and neither within the first 3 trials of a block. Also, unlike experiment 1, we did not inform the participants about any structures of the sequences.

#### Familiarity task

After listening to the sequences in both conditions in experiment 2, participants completed a familiarity test with 18 trials. In each trial, they were presented with a pair of two auditory stimuli. Their task was to categorize the pair via button press as familiar or unfamiliar. Throughout a trial, a white fixation cross was presented onscreen. The trial started with the first auditory stimulus, followed by the second auditory stimulus after one second (Fig. 3A). Four seconds after trial onset, the familiarity task instruction was displayed on the screen, asking participants to press either the left or right arrow key depending on whether the order of voices seemed familiar or unfamiliar to them. The response via key press initiated the white fixation cross display and a silent period of three seconds until the next trial started. Participants were presented with the 9 familiar stimulus pairs, i.e., the ones that participants heard in the structured condition, as well as 9 foil pairs, i.e., a random new combination of the first stimulus of each pair with the second stimulus of a different pair. The trials were presented in a randomized order.

#### Preprocessing

The preprocessing of the pupil data was performed similarly to experiment 1. As the frequencies of interest changed, we adjusted the high-pass filter to 0.25 Hz. (i.e., pass band = 0.25–5 Hz). Also, as the length of blocks changed, the Hanning window for the Welch’s method was adjusted fit one-third of a block length and the overlapping points to fit one-third of the window length. For experiment 2, data blocks were excluded when more than 33% of the pupil diameter data were missing after outlier exclusion or when participants missed more than 50% of the auditory task trials. If less than two blocks for either condition were available for analyses, the entire data set was excluded and replaced with the data set of a new participant. We excluded an average of *M* = 0.97 blocks (*SD* = 1.17, median = 0, range: 0–4) per participant in experiment 2. Excluding task trials and the trials following task trials from analyses of pupil diameter resulted in the exclusion of an average of *M* = 17.87 trials (range: 16–20) out of 81 trials per block per participant.

#### Statistical analyses

##### Main experiment

We conducted the same statistical analyses of pupil diameter for experiment 2 as for experiment 1. Due to the different timing, the analyses of spectral peaks and ITPC are based on the stimulus presentation rate at 1 Hz and pair presentation rate at 0.5 Hz. Pupil diameter maxima were identified in the time window of 200–1000 ms after stimulus onset and minima were calculated in the time window from 700 to 1500 ms after stimulus onset. We chose these time windows to prevent identifying a maximum or minimum value from the response to the preceding stimulus or ISI, as the pupil response is slightly delayed as visible in Fig. 2C.

##### Familiarity task

To check whether participants learned the pairs in the structured condition, we analysed performance and pupil diameter in the familiarity task. Performance was assessed by comparing reaction times to familiar and foil pairs by means of paired *t-*tests and by calculating whether performance accuracy was above chance. Reaction time was measured as the time in seconds of the keypress since the onscreen display of the task. We also tested whether the pupil responded differently to the familiar and foil pairs presented in the familiarity task and, thus, may serve as an indirect measure of familiarity. For this end, we preprocessed the pupil data in the same way as for the main experiment and computed the average pupil diameter per participant over all 9 trials per condition (familiar and foil pairs). Corresponding to the analyses in the experiment 1, we identified maximum pupil diameter in the 2000 ms window after onset of the second stimulus and compared maximum pupil diameter between familiar and foil pairs using a paired *t-*test. As participants could not judge the familiarity of the pair based on the first presented stimulus, but only based on the second one, we defined a 1 s-baseline before onset of the second stimulus. Additionally, to test whether participants who performed better in the familiarity task exhibited a stronger differential pupil response, we used Pearson’s correlation coefficient to measure the relationship of the mean difference in pupil diameter between foil and familiar pairs per participant with their percentage of correct categorization of the pairs.

Furthermore, we tested to which extent the pupil response reflects the actual auditory pair category and the behavioural measures of the participants (i.e., the estimated pair category as indicated by the keypress or the reaction time). To this end, we fitted a linear mixed-effects model in which we predicted the maximum pupil diameter in the familiarity task with the three fixed factors ‘true category’ (categorical; familiar = 0, foil = 1), ‘indicated category’ (categorical; familiar = 0, foil = 1) and ‘reaction time’ (continuous) as well as a random intercept for participants to account for the repeated-measures design. Note that the three fixed predictors are not correlated, as the correlation values between two predictor variables across participants do not differ from zero (true category and indicated category: mean correlation = 0.05, *t*(29) = 1.44, *p* = 0.16, *d*_*z*_ = 0.26, BF_10_ = 0.49; true category and reaction time: mean correlation = 0.08, *t*(30) = 1.83, *p* = 0.08, *d*_*z*_ = 0.33, BF_10_ = 0.84; indicated category and reaction time: mean correlation = 0.01, *t*(29) = 0.22, *p* = 0.83, *d*_*z*_ = 0.04, BF_10_ = 0.2).

## Data Availability

The datasets analysed in the current study are available in the OSF repository https://osf.io/t465x/.
